# Tris(propionitrile-κ*N*)[1,4,7-tris­(cyano­meth­yl)-1,4,7-triaza­cyclo­nonane-κ^3^ *N* ^1^,*N* ^4^,*N* ^7^]copper(II) bis­(perchlorate) dihydrate

**DOI:** 10.1107/S1600536810003211

**Published:** 2010-01-30

**Authors:** Zhong Zhang, Jianqi Lu, Difeng Wu

**Affiliations:** a College of Chemistry and Chemical Engineering, Guangxi Normal University, Yucai Road 15, Guilin 541004, People’s Republic of China

## Abstract

In the title compound, [Cu(C_3_H_5_N)_3_(C_12_H_18_N_6_)](ClO_4_)_2_·2H_2_O, the Cu^II^ atom lies on a threefold rotation axis and is coordinated in a distorted N_6_ octa­hedral environment by three tertiary amines from the tridentate chelating aza­macrocyclic ligand and three propionitrile mol­ecules. Inter­molecular non-classical C—H⋯N hydrogen bonding inter­links the [Cu(C_3_H_5_N)_3_(C_12_H_18_N_6_)]^2+^ cations into a two-dimensional supra­molecular sheet extending along the *ab* plane. The crystal packing also exhibits weak C—H⋯O inter­actions.

## Related literature

For transition metal complexes with cyano­alkyl­ated triaza­macrocycles, see: Tei *et al.* (2003 ▶). For transition metal complexes with cyano­alkyl­ated tetra­azamacrocycles, see: Aneetha *et al.* (1999 ▶); Freeman *et al.* (1984 ▶); Kang *et al.* (2002*a* ▶); Kong *et al.* (2000 ▶). For the reactivity of the pendant nitrile group attached to the aza­macrocycle, see: Freeman *et al.* (1984 ▶); Kang *et al.* (2002*b* ▶, 2005 ▶, 2008 ▶); Siegfried *et al.* (2005 ▶); Tei *et al.* (2003 ▶); Zhang *et al.* (2006 ▶). For the synthesis of the triaza­macrocyclic derivative 1,4,7-tris­(cyano­meth­yl)-1,4,7-triaza­cyclo­nonane, see: Tei *et al.* (1998 ▶).
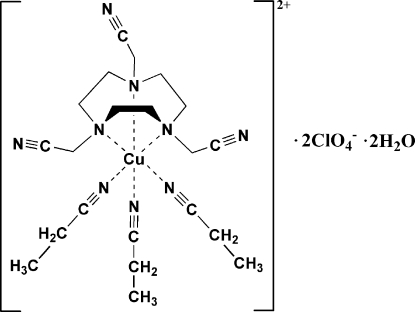


## Experimental

### 

#### Crystal data

[Cu(C_3_H_5_N)_3_(C_12_H_18_N_6_)](ClO_4_)_2_·2H_2_O
*M* *_r_* = 710.05
Trigonal, 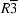

*a* = 9.962 (2) Å
*c* = 61.623 (18) Å
*V* = 5296 (2) Å^3^
*Z* = 6
Mo *K*α radiation
μ = 0.83 mm^−1^
*T* = 298 K
0.34 × 0.32 × 0.14 mm

#### Data collection

Bruker SMART APEXII CCD diffractometer
Absorption correction: multi-scan (*SADABS*; Bruker, 1998 ▶) *T*_min_ = 0.760, *T*_max_ = 0.887
9484 measured reflections
2327 independent reflections
1837 reflections with *I* > 2σ(*I*)
*R*_int_ = 0.044

#### Refinement

*R*[*F* ^2^ > 2σ(*F* ^2^)] = 0.051
*wR*(*F* ^2^) = 0.144
*S* = 0.99
2327 reflections
132 parameters
H-atom parameters constrained
Δρ_max_ = 0.26 e Å^−3^
Δρ_min_ = −0.44 e Å^−3^

### 

Data collection: *SMART* (Bruker, 2002 ▶); cell refinement: *SAINT* (Bruker, 2002 ▶); data reduction: *SAINT*; program(s) used to solve structure: *SHELXS97* (Sheldrick, 2008 ▶); program(s) used to refine structure: *SHELXL97* (Sheldrick, 2008 ▶); molecular graphics: *SHELXTL* (Sheldrick, 2008 ▶); software used to prepare material for publication: *SHELXTL*.

## Supplementary Material

Crystal structure: contains datablocks I, global. DOI: 10.1107/S1600536810003211/rk2185sup1.cif

Structure factors: contains datablocks I. DOI: 10.1107/S1600536810003211/rk2185Isup2.hkl

Additional supplementary materials:  crystallographic information; 3D view; checkCIF report

## Figures and Tables

**Table 1:** Hydrogen-bond geometry (Å, °)

*D*—H⋯*A*	*D*—H	H⋯*A*	*D*⋯*A*	*D*—H⋯*A*
C1—H1*A*⋯N2^i^	0.97	2.49	3.371 (5)	151
C3—H3*A*⋯O11	0.97	2.59	3.269 (4)	127
C6—H6*A*⋯O12^ii^	0.97	2.27	3.120 (4)	146

Symmetry codes: (i) 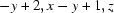; (ii) 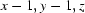.

**Fig. 1.**
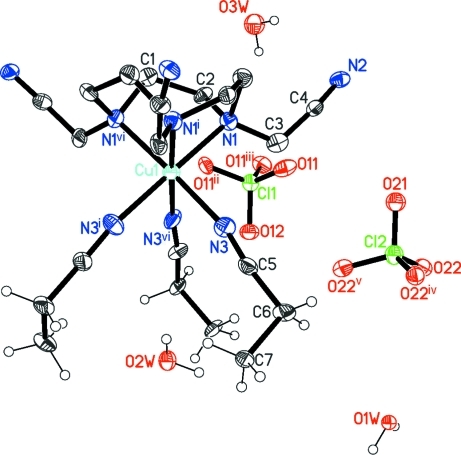
An ORTEP plot for the title compound with the atom labelling scheme. Displacement ellipsoids are drawn at 30% probability level. H atoms of the macrocyclic ligand are omitted for clarity. The disorder in the solvent water molecules are not shown. Symmetry codes: (i) -x+y+1, -x+1, z; (ii) -y+2, x-y+1, z; (iii) -x+y+1, -x+2, z; (iv) -y+1, x-y+1, z; (v) -x+y, -x+1, z; (vi) -y+1, x-y, z.

**Fig. 2.**
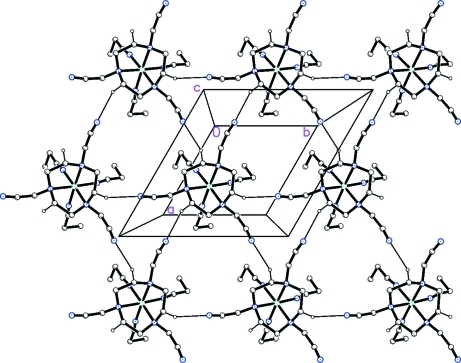
A view of the packing diagram of the title compound, showing the two–dimensional hydrogen–bonding supramolecular sheet. H atoms not involved in hydrogen bonds are omitted for clarity.

## supplementary crystallographic information

### Comment

The coordination chemistry of the azamacrocycles with nitrile pendant arms has been studied extensively. Usually, these azamacrocycle derivatives only chelate the metal through tertiary amines and the pendant nitrile groups do not involve in the coordination (Aneetha *et al.*, 1999; Freeman *et al.*, 1984; Kang *et al.*, 2002*a*; Kong *et al.*, 2000; Tei *et al.*, 2003). However, the reactivity of the nitrile group in these complexes towards nucleophilic reagents, such as water, alcohols and amines, provides a convenient route to the synthesis of a variety of *N*–functionalized azamacrocycles (Freeman *et al.*, 1984; Kang *et al.*, 2002*b*, 2005, 2008; Siegfried *et al.*, 2005; Tei *et al.*, 2003; Zhang *et al.*, 2006). In order to obtain further knowledge about the reactivity of the nitrile groups attached to the triazamacrocycle, a title compound of Cu^II^ with 1,4,7–tris(cyanomethyl)–1,4,7–triazacyclononane has been prepared and structurally characterized.

As shown in Fig. 1, the distorted octahedral Cu^II^ center in the title compound is located on a threefold rotation axis and is ligated by three N donors of the tridentate azamacrocycle backbone and other three from coordinated propionitrile molecules. The Cu—N(macrocycle) length (2.089 (3)Å) is slightly longer than that of the Cu—N(propionitrile) (2.030 (3)Å), while the bond angles subtended by *cis*–pairs of donor atoms at Cu^II^ range from 84.13 (13)° to 96.12 (11)°. In the *ab*–plane, each [Cu(C_12_H_18_N_6_)(C_3_H_5_N)_3_]^2+^ cation are linked with six neighbouring cations by means of C—H···N hydrogen bonding (Table 1) to form an extended two–dimensional supramolecular network, as depicted in Fig. 2. Perchlorate counter–anions are embedded in the two–dimensional supramolecular cationic layer *via* weak interactions.

### Experimental

The triazamacrocyclic derivative 1,4,7–tris(cyanomethyl)–1,4,7–triazacyclononane was prepared according to a published method (Tei *et al.*, 1998).

To the propionitrile solution (20 ml) of the triazamacrocyclic ligand (49 mg, 0.2 mmol), Cu(ClO_4_)_2_^.^6H_2_O (74 mg, 0.2 mmol) was added. The resulting mixture was stirred under reflux for 4 h and then cooled to ambient temperature. Blue single crystals of title compound suitable for X–ray diffraction analysis were obtained by slow diffusion of diethyl ether into the complex solution. (yield 51 mg, 72%). Elemental analysis found: C 35.52; H 5.39; N 17.69%; calculated for C_21_H_37_Cl_2_CuN_9_O_10_: C 35.31; H 5.28; N 17.75%.

### Refinement

All H atoms were placed in calculated positions and refined as riding atoms, with C—H = 0.96–0.97Å and O—H = 0.85Å, and with *U*_iso_(H) = 1.2*U*_eq_(C and O) or 1.5*U*_eq_(methyl C). OW2 lies on a threefold rotation axis, so its hydrogen atoms are disordered with site occupancy factor of 0.33. OW1 and OW3 are disordered on special positions with threefold roto–inversion symmetry and each of them has 0.17 occupancy in the asymmetric unit.

### Crystal data

**Table:**

[Cu(C_3_H_5_N)_3_(C_12_H_18_N_6_)](ClO_4_)_2_·2H_2_O	*D*_x_ = 1.336 Mg m^−^^3^
*M**_r_* = 710.05	Mo *K*α radiation, λ = 0.71073 Å
Trigonal, *R*3	Cell parameters from 1799 reflections
Hall symbol: -R 3	θ = 2.4–20.9°
*a* = 9.962 (2) Å	µ = 0.83 mm^−^^1^
*c* = 61.623 (18) Å	*T* = 298 K
*V* = 5296 (2) Å^3^	Plate, blue
*Z* = 6	0.34 × 0.32 × 0.14 mm
*F*(000) = 2214	

### Data collection

**Table:**

Bruker SMART APEXII CCD diffractometer	2327 independent reflections
Radiation source: fine–focus sealed tube	1837 reflections with *I* > 2σ(*I*)
graphite	*R*_int_ = 0.044
φ and ω scans	θ_max_ = 26.0°, θ_min_ = 2.0°
Absorption correction: multi-scan (*SADABS*; Bruker, 1998)	*h* = −12→12
*T*_min_ = 0.760, *T*_max_ = 0.887	*k* = −7→12
9484 measured reflections	*l* = −75→64

### Refinement

**Table:**

Refinement on *F*^2^	Primary atom site location: structure-invariant direct methods
Least-squares matrix: full	Secondary atom site location: difference Fourier map
*R*[*F*^2^ > 2σ(*F*^2^)] = 0.051	Hydrogen site location: inferred from neighbouring sites
*wR*(*F*^2^) = 0.144	H-atom parameters constrained
*S* = 0.99	*w* = 1/[σ^2^(*F*_o_^2^) + (0.0959*P*)^2^ + 1.480*P*] where *P* = (*F*_o_^2^ + 2*F*_c_^2^)/3
2327 reflections	(Δ/σ)_max_ < 0.001
132 parameters	Δρ_max_ = 0.26 e Å^−^^3^
0 restraints	Δρ_min_ = −0.44 e Å^−^^3^

### Special details

**Table:**

Geometry. All s.u.'s (except the s.u. in the dihedral angle between two l.s. planes) are estimated using the full covariance matrix. The cell s.u.'s are taken into account individually in the estimation of s.u.'s in distances, angles and torsion angles; correlations between s.u.'s in cell parameters are only used when they are defined by crystal symmetry. An approximate (isotropic) treatment of cell s.u.'s is used for estimating s.u.'s involving l.s. planes.
Refinement. Refinement of *F*^2^ against ALL reflections. The weighted *R*–factor wR and goodness of fit *S* are based on *F*^2^, conventional *R*–factors *R* are based on *F*, with *F* set to zero for negative *F*^2^. The threshold expression of *F*^2^ > σ(*F*^2^) is used only for calculating *R*–factors(gt) etc. and is not relevant to the choice of reflections for refinement. *R*–factors based on *F*^2^ are statistically about twice as large as those based on *F*, and *R*–factors based on ALL data will be even larger.

### Fractional atomic coordinates and isotropic or equivalent isotropic displacement parameters (Å^2^)

**Table:**

	*x*	*y*	*z*	*U*_iso_*/*U*_eq_	Occ. (<1)
C1	0.8592 (4)	0.4740 (4)	0.02573 (6)	0.0412 (8)	
H1A	0.9682	0.5407	0.0224	0.049*	
H1B	0.8078	0.4188	0.0126	0.049*	
C2	0.7901 (4)	0.5749 (4)	0.03281 (5)	0.0336 (7)	
H2A	0.8615	0.6547	0.0427	0.040*	
H2B	0.7778	0.6258	0.0202	0.040*	
C3	0.5888 (4)	0.5827 (4)	0.05172 (5)	0.0379 (8)	
H3A	0.6568	0.6427	0.0635	0.045*	
H3B	0.4873	0.5155	0.0580	0.045*	
C4	0.5751 (4)	0.6906 (4)	0.03705 (5)	0.0360 (7)	
C5	0.4092 (4)	0.2743 (4)	0.09939 (5)	0.0394 (8)	
C6	0.3004 (4)	0.2578 (4)	0.11629 (5)	0.0389 (8)	
H6A	0.1959	0.2111	0.1105	0.047*	
H6B	0.3281	0.3578	0.1225	0.047*	
C7	0.3123 (5)	0.1525 (4)	0.13332 (5)	0.0404 (8)	
H7A	0.3651	0.1029	0.1272	0.061*	
H7B	0.2101	0.0751	0.1378	0.061*	
H7C	0.3691	0.2133	0.1456	0.061*	
Cl1	1.0000	1.0000	0.07171 (2)	0.0373 (3)	
Cl2	0.3333	0.6667	0.09665 (2)	0.0383 (3)	
Cu1	0.6667	0.3333	0.063982 (10)	0.0316 (2)	
N1	0.6443 (3)	0.4868 (3)	0.04321 (4)	0.0335 (6)	
N2	0.5838 (3)	0.7880 (3)	0.02473 (4)	0.0385 (7)	
N3	0.4964 (4)	0.3039 (3)	0.08486 (5)	0.0423 (7)	
O1	0.3333	0.6667	0.1667	0.0247 (10)	
H1C	0.3300	0.6081	0.1769	0.037*	0.16667
H1D	0.3867	0.7608	0.1706	0.037*	0.16667
O2	0.6667	0.3333	0.14060 (6)	0.0353 (8)	
H2C	0.6152	0.3741	0.1454	0.042*	0.33333
H2D	0.6160	0.2696	0.1305	0.042*	0.33333
O3	1.0000	1.0000	0.0000	0.0544 (16)	
H3D	1.0241	1.0481	0.0120	0.065*	0.16667
H3C	0.9020	0.9506	−0.0014	0.082*	0.16667
O11	0.8501 (3)	0.9383 (3)	0.06411 (4)	0.0473 (7)	
O12	1.0000	1.0000	0.09409 (6)	0.0442 (10)	
O21	0.3333	0.6667	0.07487 (7)	0.0413 (10)	
O22	0.3579 (3)	0.8074 (3)	0.10312 (4)	0.0379 (5)	

### Atomic displacement parameters (Å^2^)

**Table:**

	*U*^11^	*U*^22^	*U*^33^	*U*^12^	*U*^13^	*U*^23^
C1	0.0410 (19)	0.0400 (19)	0.0380 (17)	0.0170 (16)	0.0013 (14)	−0.0008 (14)
C2	0.0297 (15)	0.0339 (16)	0.0334 (15)	0.0131 (13)	0.0009 (12)	−0.0114 (13)
C3	0.0303 (16)	0.0250 (15)	0.0417 (17)	0.0014 (13)	0.0066 (13)	0.0010 (13)
C4	0.0364 (17)	0.0447 (18)	0.0314 (15)	0.0238 (15)	0.0036 (13)	−0.0041 (14)
C5	0.0386 (19)	0.0444 (19)	0.0345 (16)	0.0203 (16)	−0.0006 (14)	0.0139 (14)
C6	0.0439 (19)	0.0317 (17)	0.0335 (16)	0.0132 (15)	0.0138 (14)	0.0150 (13)
C7	0.056 (2)	0.0431 (19)	0.0344 (16)	0.0335 (18)	0.0156 (15)	0.0193 (15)
Cl1	0.0360 (5)	0.0360 (5)	0.0400 (7)	0.0180 (2)	0.000	0.000
Cl2	0.0363 (5)	0.0363 (5)	0.0421 (7)	0.0182 (2)	0.000	0.000
Cu1	0.0322 (3)	0.0322 (3)	0.0304 (4)	0.01610 (14)	0.000	0.000
N1	0.0323 (14)	0.0296 (13)	0.0363 (14)	0.0138 (11)	0.0006 (11)	0.0048 (10)
N2	0.0368 (15)	0.0411 (16)	0.0304 (13)	0.0142 (13)	0.0070 (11)	0.0034 (12)
N3	0.0474 (17)	0.0317 (15)	0.0431 (15)	0.0163 (13)	0.0109 (14)	0.0150 (12)
O1	0.0253 (15)	0.0253 (15)	0.023 (2)	0.0126 (7)	0.000	0.000
O2	0.0318 (12)	0.0318 (12)	0.042 (2)	0.0159 (6)	0.000	0.000
O3	0.054 (2)	0.054 (2)	0.054 (4)	0.0272 (12)	0.000	0.000
O11	0.0335 (13)	0.0445 (14)	0.0399 (13)	0.0015 (11)	0.0118 (10)	−0.0034 (11)
O12	0.0503 (16)	0.0503 (16)	0.032 (2)	0.0252 (8)	0.000	0.000
O21	0.0424 (15)	0.0424 (15)	0.039 (2)	0.0212 (7)	0.000	0.000
O22	0.0341 (12)	0.0427 (13)	0.0341 (11)	0.0172 (11)	−0.0008 (9)	0.0054 (10)

### Geometric parameters (Å, °)

**Table:**

C1—C2	1.538 (5)	Cl1—O11^ii^	1.382 (3)
C1—N1^i^	1.543 (4)	Cl1—O11	1.382 (3)
C1—H1A	0.9700	Cl1—O11^iii^	1.382 (3)
C1—H1B	0.9700	Cl2—O21	1.342 (4)
C2—N1	1.420 (4)	Cl2—O22	1.357 (3)
C2—H2A	0.9700	Cl2—O22^iv^	1.357 (3)
C2—H2B	0.9700	Cl2—O22^v^	1.357 (3)
C3—N1	1.422 (5)	Cu1—N3^vi^	2.030 (3)
C3—C4	1.462 (5)	Cu1—N3^i^	2.030 (3)
C3—H3A	0.9700	Cu1—N3	2.030 (3)
C3—H3B	0.9700	Cu1—N1^vi^	2.089 (3)
C4—N2	1.200 (5)	Cu1—N1^i^	2.089 (3)
C5—N3	1.178 (5)	Cu1—N1	2.089 (3)
C5—C6	1.452 (5)	N1—C1^vi^	1.543 (4)
C6—C7	1.529 (4)	O1—H1C	0.8500
C6—H6A	0.9700	O1—H1D	0.8500
C6—H6B	0.9700	O2—H2C	0.8500
C7—H7A	0.9600	O2—H2D	0.8500
C7—H7B	0.9600	O3—H3D	0.8500
C7—H7C	0.9600	O3—H3C	0.8500
Cl1—O12	1.379 (4)		
			
C2—C1—N1^i^	112.9 (3)	O12—Cl1—O11^iii^	109.83 (11)
C2—C1—H1A	109.0	O11^ii^—Cl1—O11^iii^	109.11 (11)
N1^i^—C1—H1A	109.0	O11—Cl1—O11^iii^	109.11 (11)
C2—C1—H1B	109.0	O21—Cl2—O22	107.08 (11)
N1^i^—C1—H1B	109.0	O21—Cl2—O22^iv^	107.08 (11)
H1A—C1—H1B	107.8	O22—Cl2—O22^iv^	111.75 (10)
N1—C2—C1	112.2 (3)	O21—Cl2—O22^v^	107.08 (11)
N1—C2—H2A	109.2	O22—Cl2—O22^v^	111.75 (10)
C1—C2—H2A	109.2	O22^iv^—Cl2—O22^v^	111.75 (10)
N1—C2—H2B	109.2	N3^vi^—Cu1—N3^i^	84.13 (13)
C1—C2—H2B	109.2	N3^vi^—Cu1—N3	84.13 (13)
H2A—C2—H2B	107.9	N3^i^—Cu1—N3	84.13 (13)
N1—C3—C4	118.5 (3)	N3^vi^—Cu1—N1^vi^	96.12 (11)
N1—C3—H3A	107.7	N3^i^—Cu1—N1^vi^	177.46 (11)
C4—C3—H3A	107.7	N3—Cu1—N1^vi^	93.37 (12)
N1—C3—H3B	107.7	N3^vi^—Cu1—N1^i^	93.37 (12)
C4—C3—H3B	107.7	N3^i^—Cu1—N1^i^	96.12 (11)
H3A—C3—H3B	107.1	N3—Cu1—N1^i^	177.46 (11)
N2—C4—C3	171.7 (4)	N1^vi^—Cu1—N1^i^	86.40 (11)
N3—C5—C6	171.5 (4)	N3^vi^—Cu1—N1	177.46 (11)
C5—C6—C7	105.1 (3)	N3^i^—Cu1—N1	93.37 (12)
C5—C6—H6A	110.7	N3—Cu1—N1	96.12 (11)
C7—C6—H6A	110.7	N1^vi^—Cu1—N1	86.40 (11)
C5—C6—H6B	110.7	N1^i^—Cu1—N1	86.40 (11)
C7—C6—H6B	110.7	C2—N1—C3	111.9 (2)
H6A—C6—H6B	108.8	C2—N1—C1^vi^	107.5 (2)
C6—C7—H7A	109.5	C3—N1—C1^vi^	105.8 (3)
C6—C7—H7B	109.5	C2—N1—Cu1	106.2 (2)
H7A—C7—H7B	109.5	C3—N1—Cu1	119.0 (2)
C6—C7—H7C	109.5	C1^vi^—N1—Cu1	105.87 (19)
H7A—C7—H7C	109.5	C5—N3—Cu1	167.5 (3)
H7B—C7—H7C	109.5	H1C—O1—H1D	109.4
O12—Cl1—O11^ii^	109.83 (11)	H2C—O2—H2D	109.5
O12—Cl1—O11	109.83 (11)	H3D—O3—H3C	109.5
O11^ii^—Cl1—O11	109.11 (11)		
			
N1^i^—C1—C2—N1	−43.3 (4)	N3—Cu1—N1—C3	28.2 (2)
C1—C2—N1—C3	173.9 (3)	N1^vi^—Cu1—N1—C3	121.2 (3)
C1—C2—N1—C1^vi^	−70.5 (4)	N1^i^—Cu1—N1—C3	−152.2 (3)
C1—C2—N1—Cu1	42.5 (3)	N3^i^—Cu1—N1—C1^vi^	−175.0 (2)
C4—C3—N1—C2	54.9 (4)	N3—Cu1—N1—C1^vi^	−90.6 (2)
C4—C3—N1—C1^vi^	−61.8 (3)	N1^vi^—Cu1—N1—C1^vi^	2.44 (19)
C4—C3—N1—Cu1	179.4 (2)	N1^i^—Cu1—N1—C1^vi^	89.05 (15)
N3^i^—Cu1—N1—C2	71.0 (2)	N3^vi^—Cu1—N3—C5	10.1 (14)
N3—Cu1—N1—C2	155.4 (2)	N3^i^—Cu1—N3—C5	−74.6 (13)
N1^vi^—Cu1—N1—C2	−111.58 (15)	N1^vi^—Cu1—N3—C5	105.9 (14)
N1^i^—Cu1—N1—C2	−25.0 (2)	N1—Cu1—N3—C5	−167.3 (14)
N3^i^—Cu1—N1—C3	−56.3 (2)		

Symmetry codes: (i) −*x*+*y*+1, −*x*+1, *z*; (ii) −*y*+2, *x*−*y*+1, *z*; (iii) −*x*+*y*+1, −*x*+2, *z*; (iv) −*y*+1, *x*−*y*+1, *z*; (v) −*x*+*y*, −*x*+1, *z*; (vi) −*y*+1, *x*−*y*, *z*.

### Hydrogen-bond geometry (Å, °)

**Table:**

*D*—H···*A*	*D*—H	H···*A*	*D*···*A*	*D*—H···*A*
C1—H1A···N2^ii^	0.97	2.49	3.371 (5)	151
C3—H3A···O11	0.97	2.59	3.269 (4)	127
C6—H6A···O12^vii^	0.97	2.27	3.120 (4)	146

Symmetry codes: (ii) −*y*+2, *x*−*y*+1, *z*; (vii) *x*−1, *y*−1, *z*.
